# Supplementary figures and images for: Computational prediction of protein interactions in single cells by proximity sequencing
Source: PLoS Comput Biol. 2024 Mar 14;20(3):e1011915. doi: 10.1371/journal.pcbi.1011915 (PMC10939233; doi:10.1371/journal.pcbi.1011915)

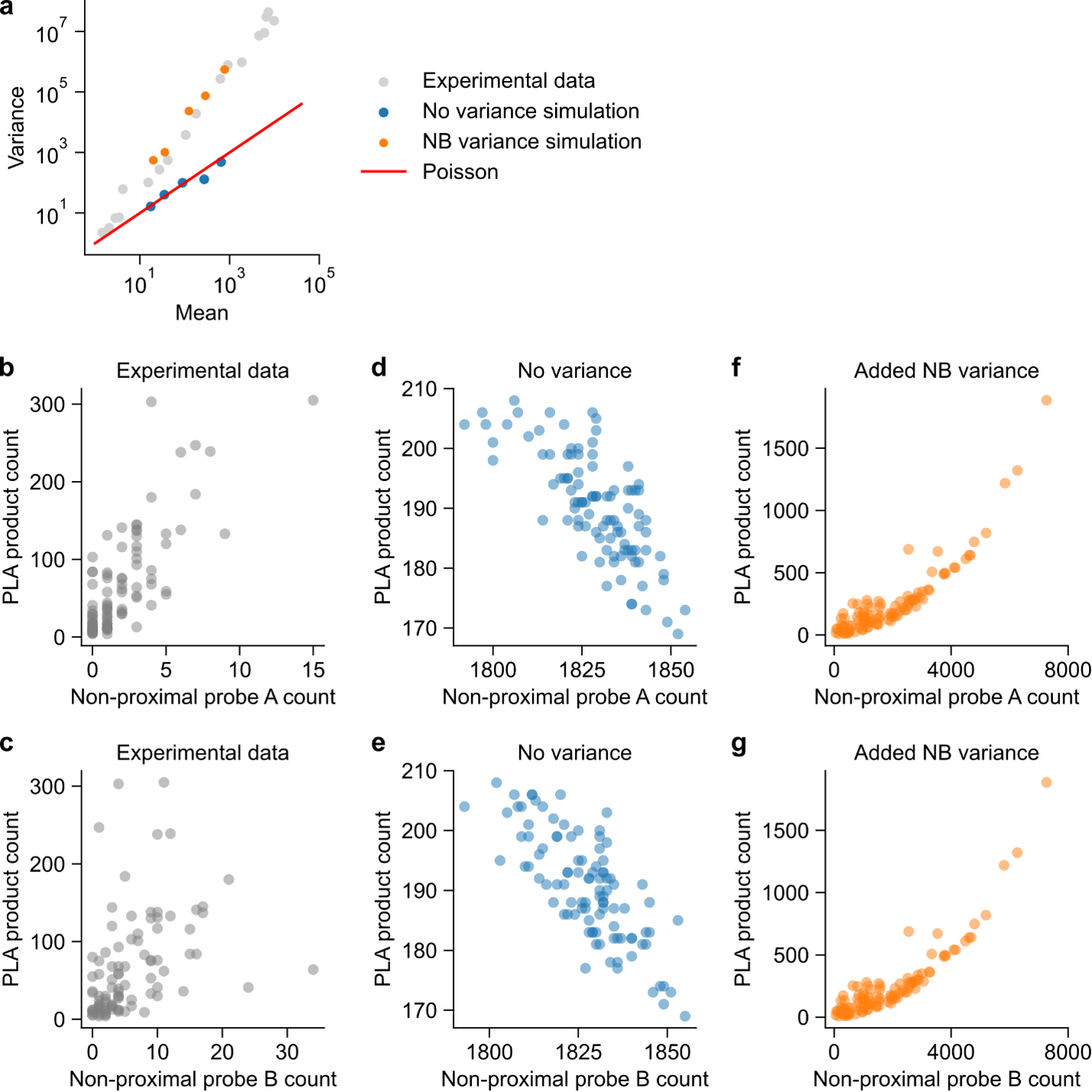

Supplement: S1 Fig — (a) Scatter plot showing the mean-variance relationship in real and simulated protein count. (b, c) Scatter plots showing the relationship between observed CD3:CD3 PLA product and (b) non-proximal CD3 probe A or (c) non-proximal CD3 probe B in Jurkat cells. (d, e) Scatter plots showing the relationship between observed 1:1 PLA product and (d) non-proximal protein 1 probe A or (e) non-proximal protein 1 probe B in simulated data without variance. (f, g) Scatter plots showing the relationship between observed 1:1 PLA product and (f) non-proximal protein 1 probe A or (g) non-proximal protein 1 probe B in simulated data with negative binomial variance. (TIF) [file pcbi.1011915.s001.tif]

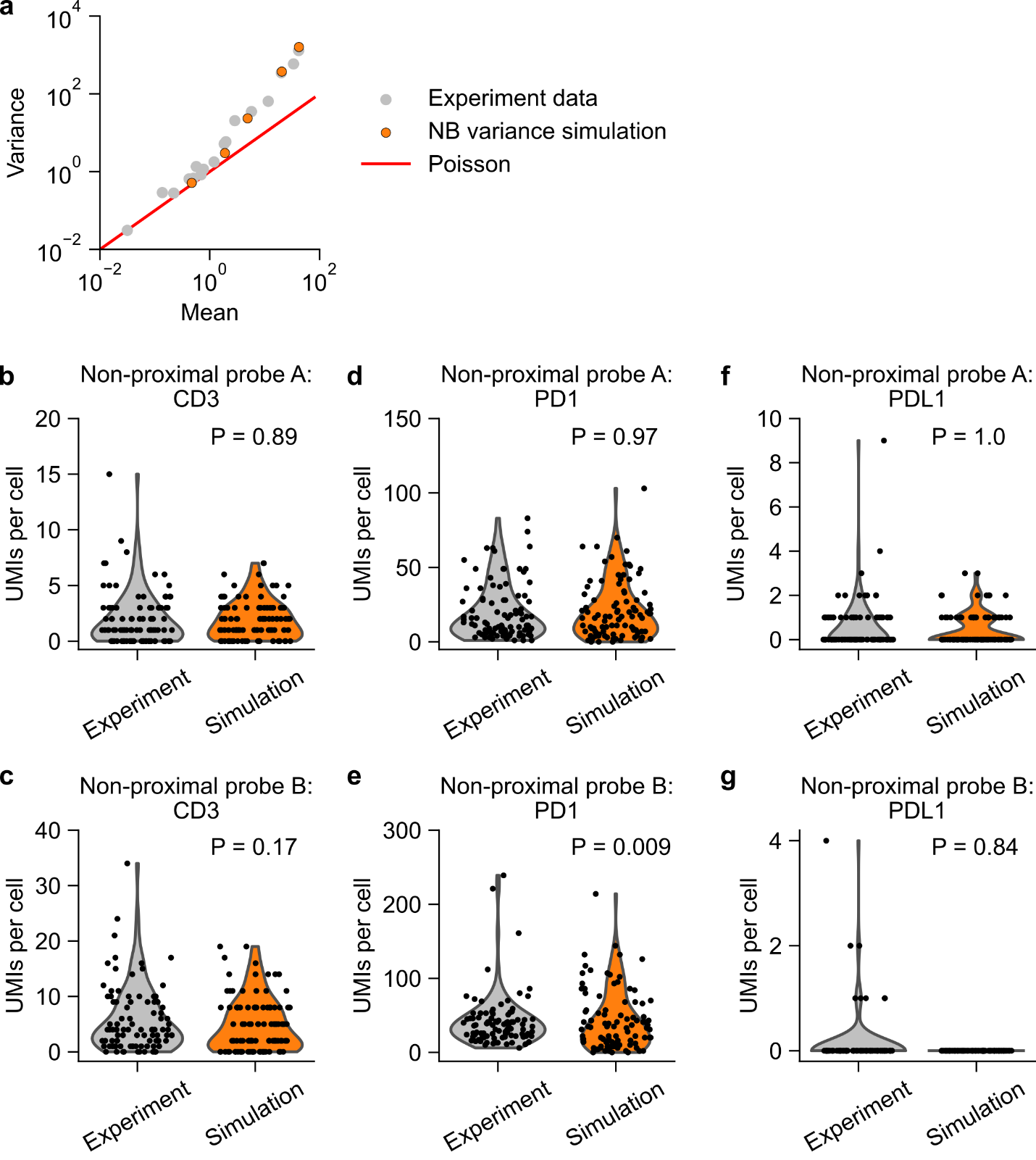

Supplement: S2 Fig — (a) Scatter plot showing the mean-variance relationship in experimental (Jurkat cells) and simulated non-proximal probe count. (b, c) Violin plots showing the experimental and simulated count of (b) non-proximal probe A and (c) non-proximal probe B for CD3 protein. (d, e) Violin plots showing the experimental and simulated count of (d) non-proximal probe A and (e) non-proximal probe B for PD1 protein. (f, g) Violin plots showing the experimental and simulated count of (f) non-proximal probe A and (g) non-proximal probe B for PDL1 protein. non-proximal probe counts for CD3, PD1 and PDL1 proteins were simulated by using the mean non-proximal probe counts in experimental data. Note that Jurkat cells expressed CD3 and PD1 proteins, but not PDL1 protein. P-values are calculated using KS test. (TIF) [file pcbi.1011915.s002.tif]

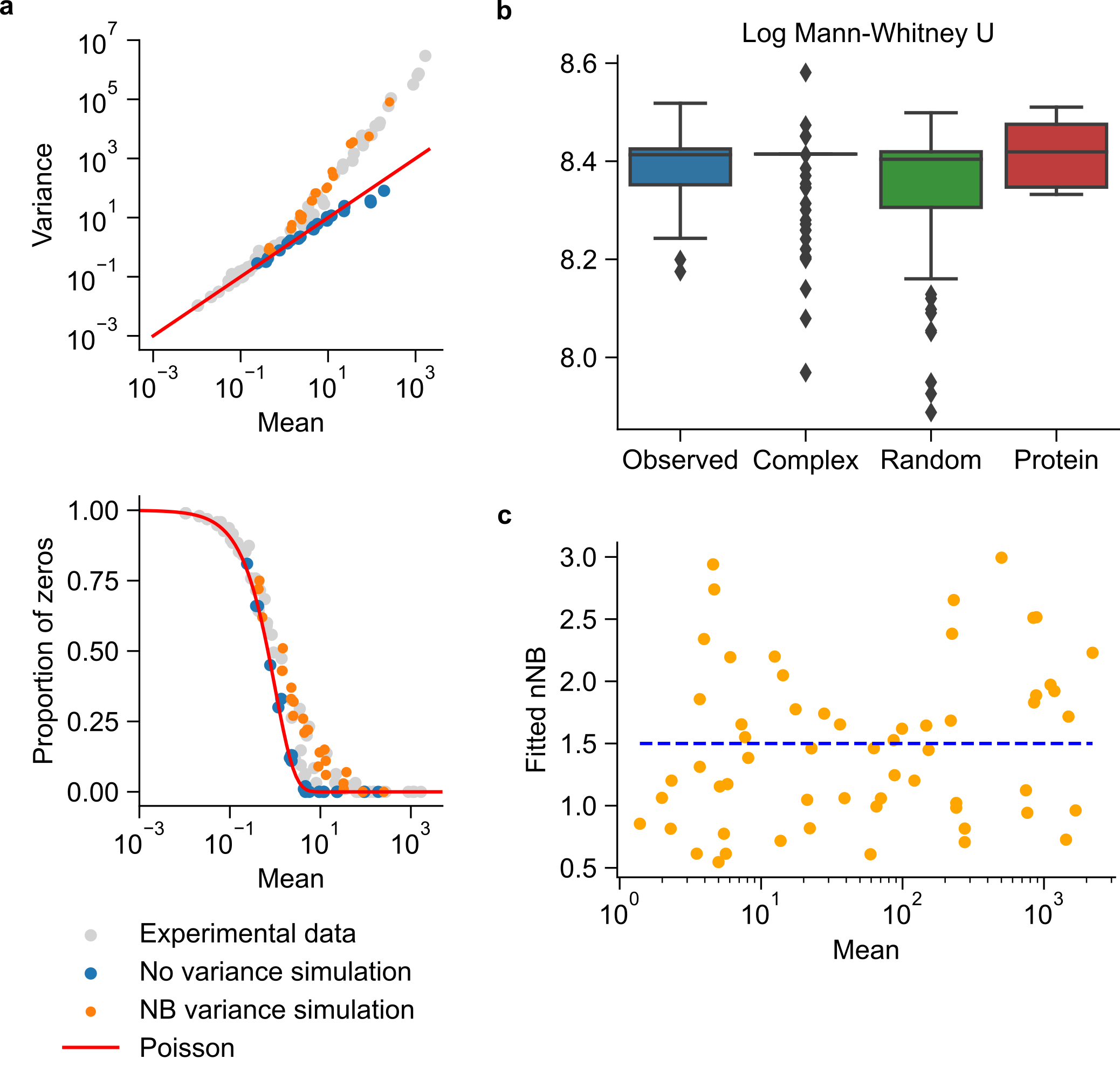

Supplement: S3 Fig — (a) The simulation with NB variance outperforms Poisson variance for both the mean-variance relationship (top) and proportion of zeroes (bottom). (b) For each observed PLA, protein complex, proximity noise and protein, the Mann-Whitney U statistic between posterior predictive samples and observed data averaged over samples. Box plots indicate the median (center line), interquartile range (hinges), and whiskers at 1.5x interquartile range. Higher is better. (c) Scatter plot of fitted nNB value versus mean value of PLA products on Jurkat and Raji cells. PLA product count is fit with a negative binomial distribution model across single cells. Different PLA products have different best-fit nNB values. As shown by the dashed blue horizontal line, the mean value of nNB is close to 1.5 for PLA products of different mean values. Thus, nNB = 1.5 is used as default value in the simulation model. (TIF) [file pcbi.1011915.s003.tif]

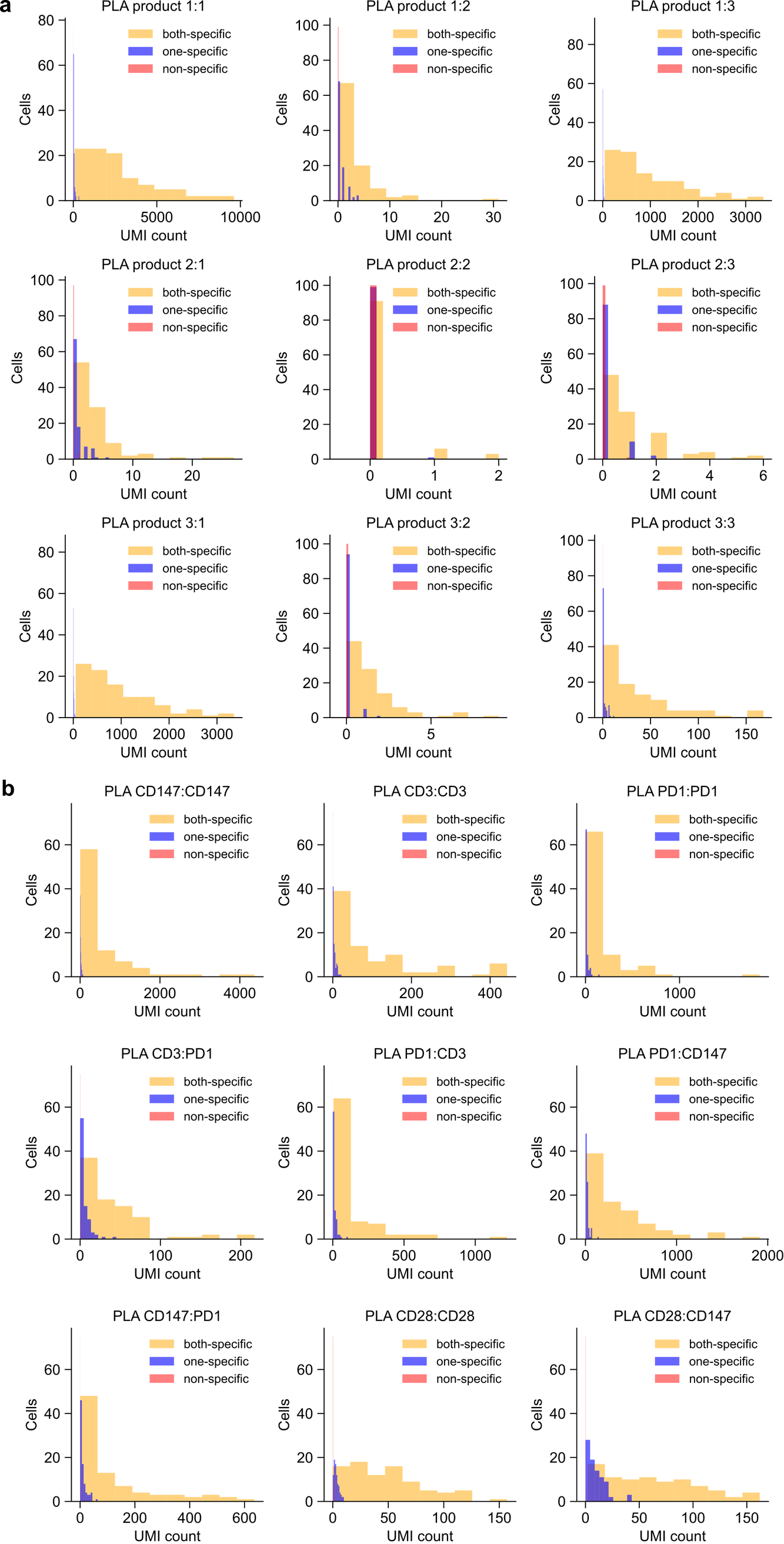

Supplement: S4 Fig — (a) Simulation data where nonspecific binding probability of antibody 1, 2, 3 is set to 0.2, 0.1, and 0.05, respectively. (b) Experimental data where isotype control antibody is used to estimate the probability of nonspecific binding. (TIF) [file pcbi.1011915.s004.tif]

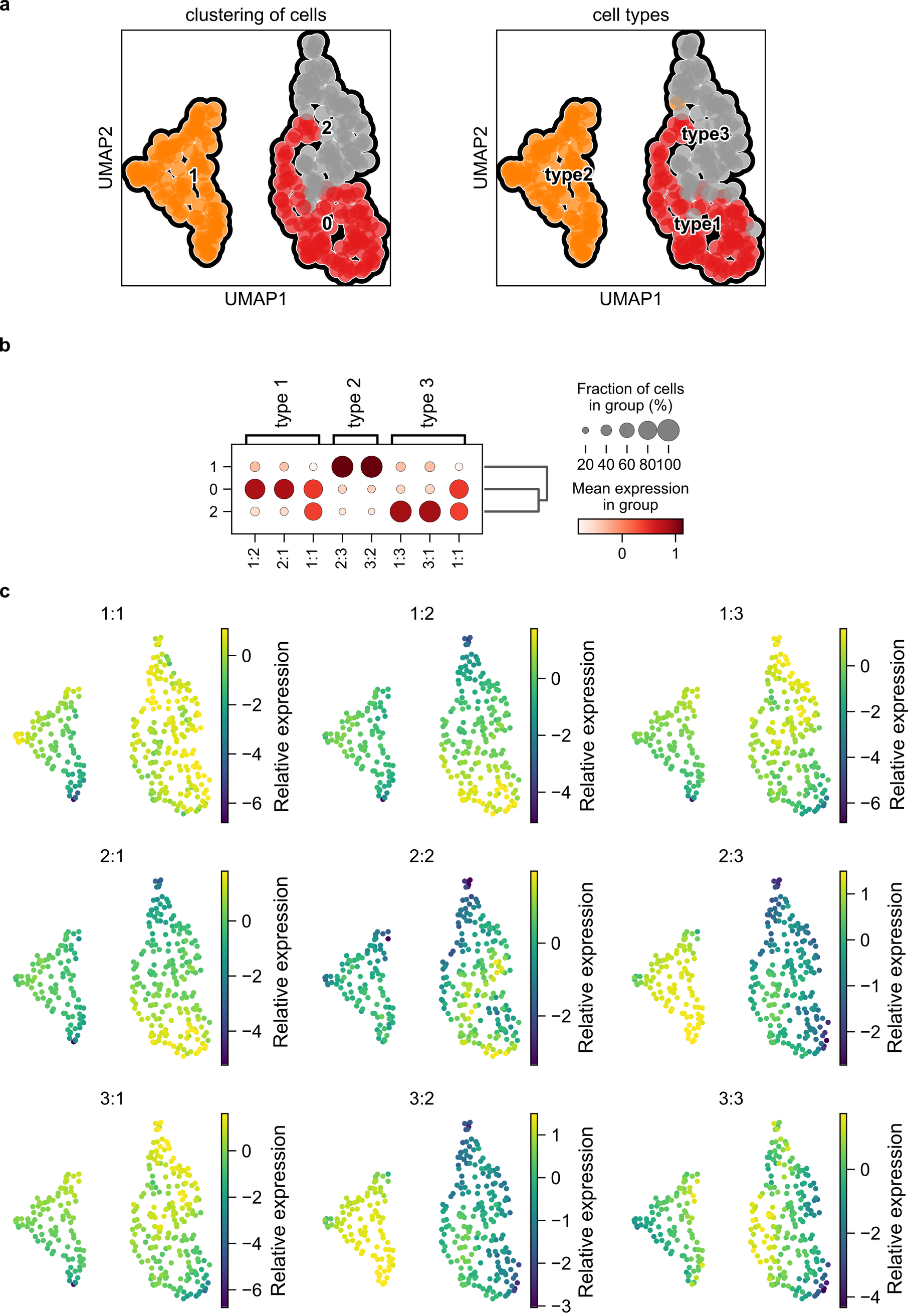

Supplement: S5 Fig — (a) Single cell uniform manifold approximation and projection (UMAP) plot of three cell types. Clustering of cells is computationally determined by unsupervised learning. Cell types are ground truth for comparison. (b) Dot plot showing that differential expression analysis of each cluster caputures the featured PLA product of each cell type. (c) UMAP plot showing the relative expression (log2FC) of all PLA products in each cell cluster. (TIF) [file pcbi.1011915.s005.tif]

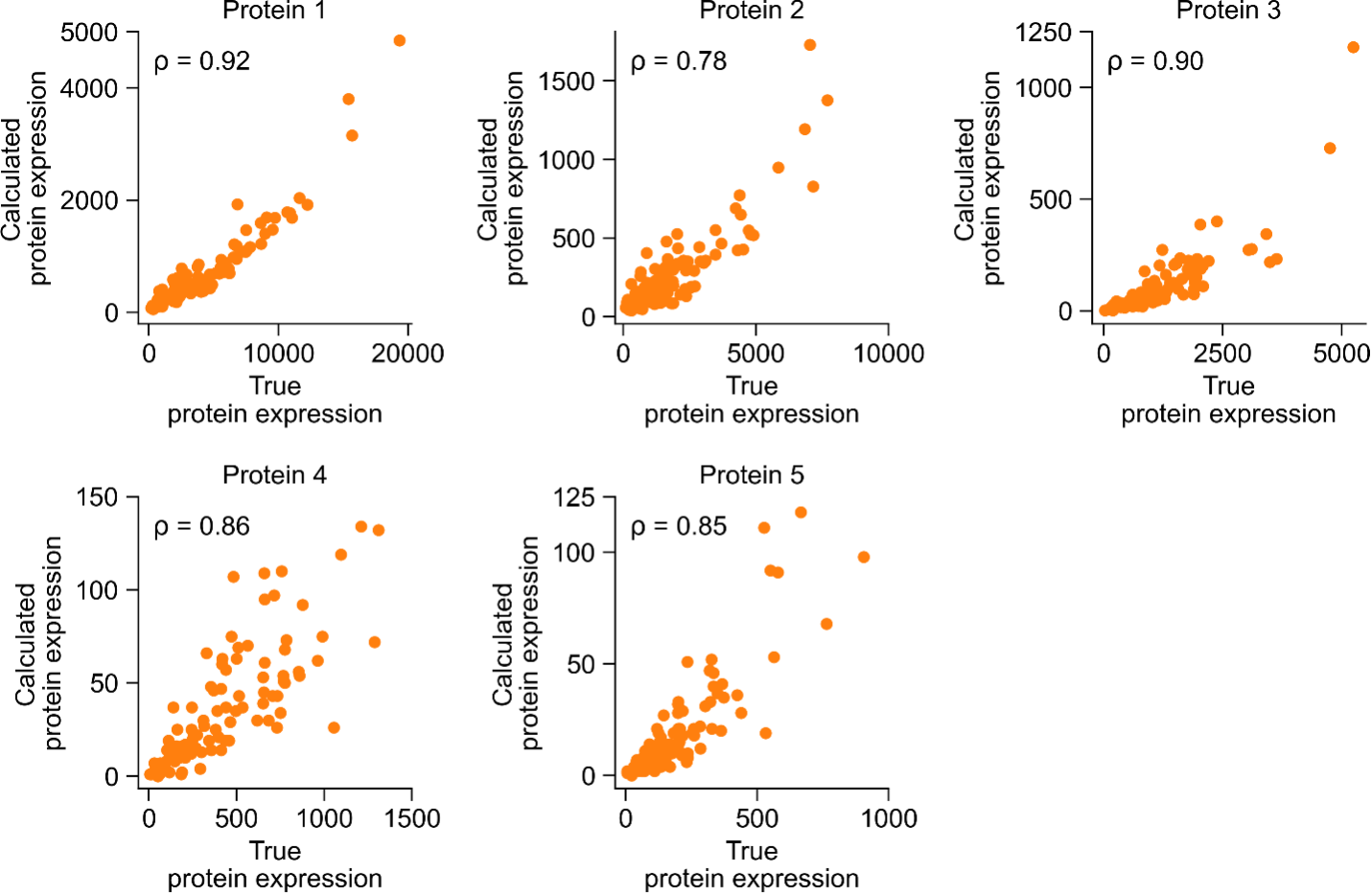

Supplement: S6 Fig — Scatter plots showing the correlation between true and calculated protein expression in simulated data. The protein expression is equal to the UMI count of each protein per single cell. Each panel also displays the corresponding Spearman’s correlation coefficient, ρ. (TIF) [file pcbi.1011915.s006.tif]

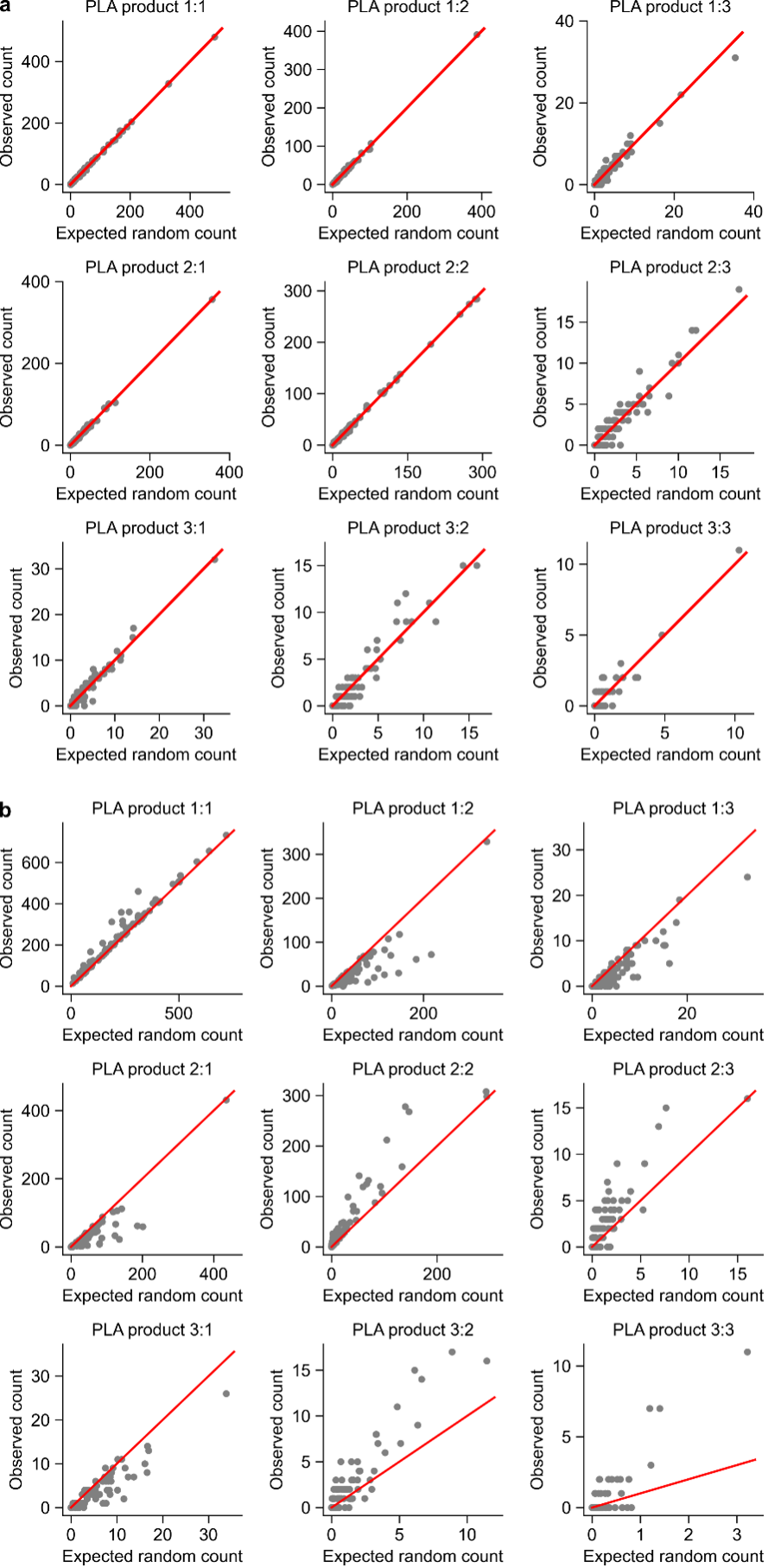

Supplement: S7 Fig — (a, b) Scatter plots showing the observed and expected random count of each PLA product in the scenario when (a) no protein complex, and when (b) 1:1 is the only protein complex. The red lines indicate y = x. (TIF) [file pcbi.1011915.s007.tif]

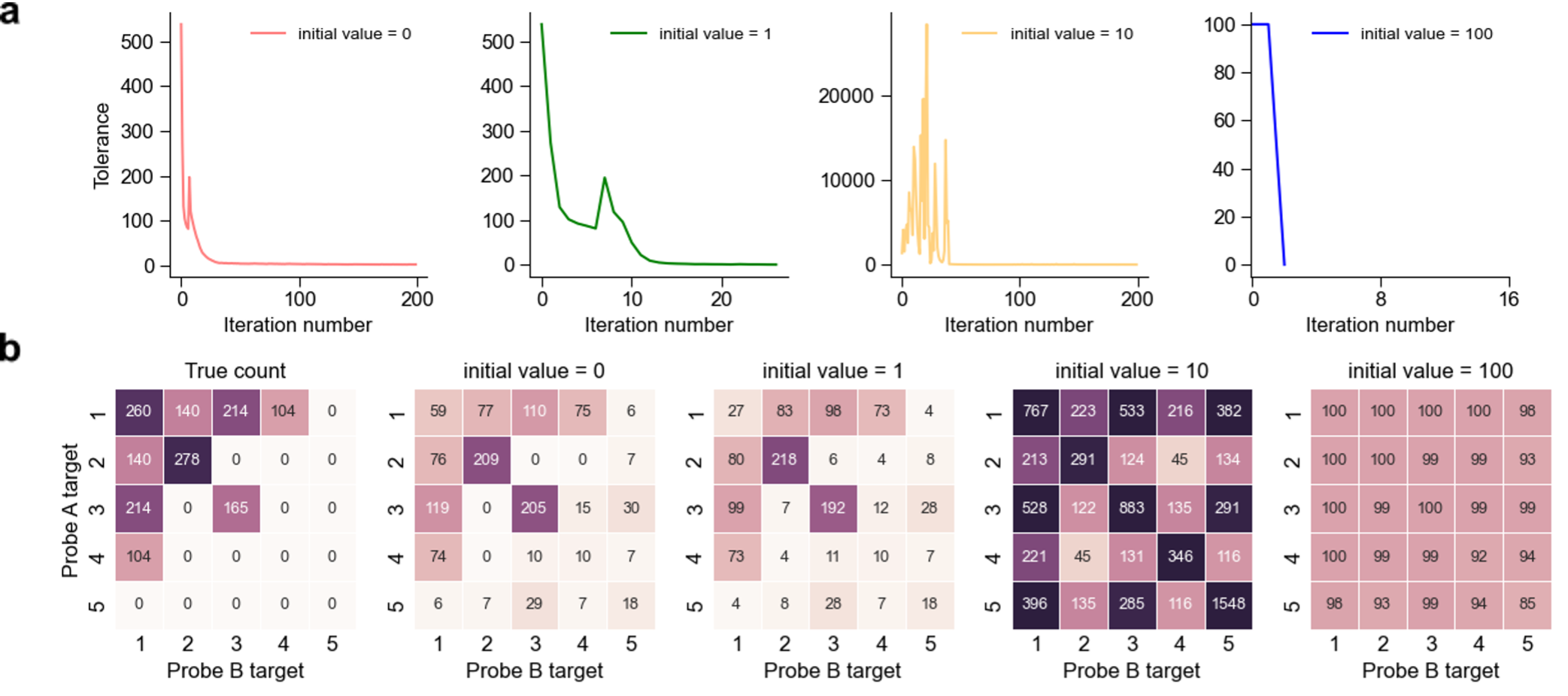

Supplement: S8 Fig — (a) The change in output for each iteration (tolerance) changes depending on the initial values given to the algorithm. (b) The resulting protein complex estimates for each initialization, compared to the true complex values (left panel). (TIF) [file pcbi.1011915.s008.tif]

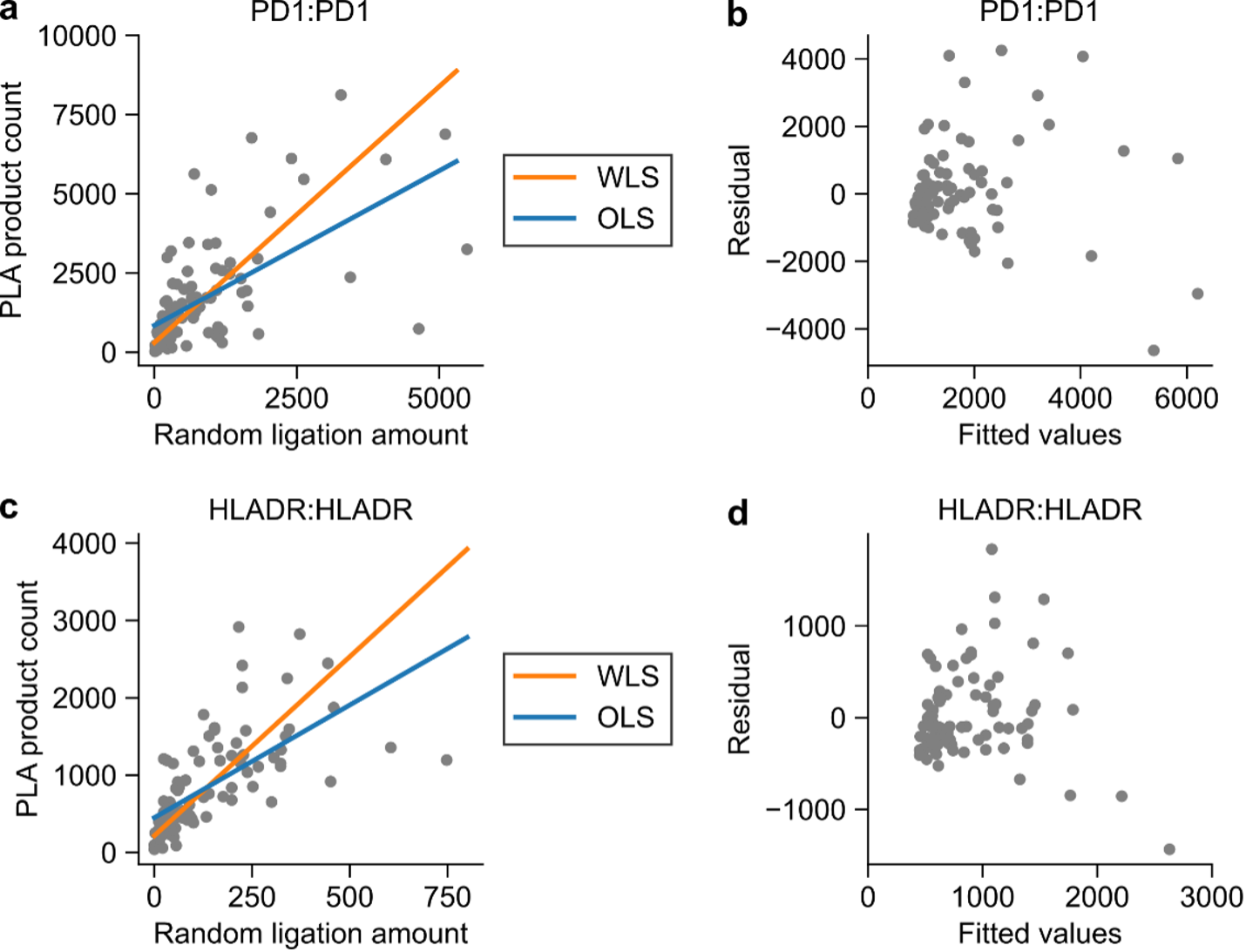

Supplement: S9 Fig — (a) Scatter plot showing the relationship between observed count of PLA product PD1:PD1 and the measured random ligation amount in Jurkat cells, and the corresponding weighted least squares (WLS) and ordinary least squares (OLS) regression lines. (b) Residual plot of ordinary least squares regression for PLA product PD1:PD1 in Jurkat cells. (c) Scatter plot showing the relationship between observed count of PLA product HLADR:HLADR and the measured random ligation amount in Raji cells, and the corresponding WLS and OLS regression lines. (d) Residual plot of ordinary least squares regression for PLA product HLADR:HLADR in Raji cells. (TIF) [file pcbi.1011915.s009.tif]

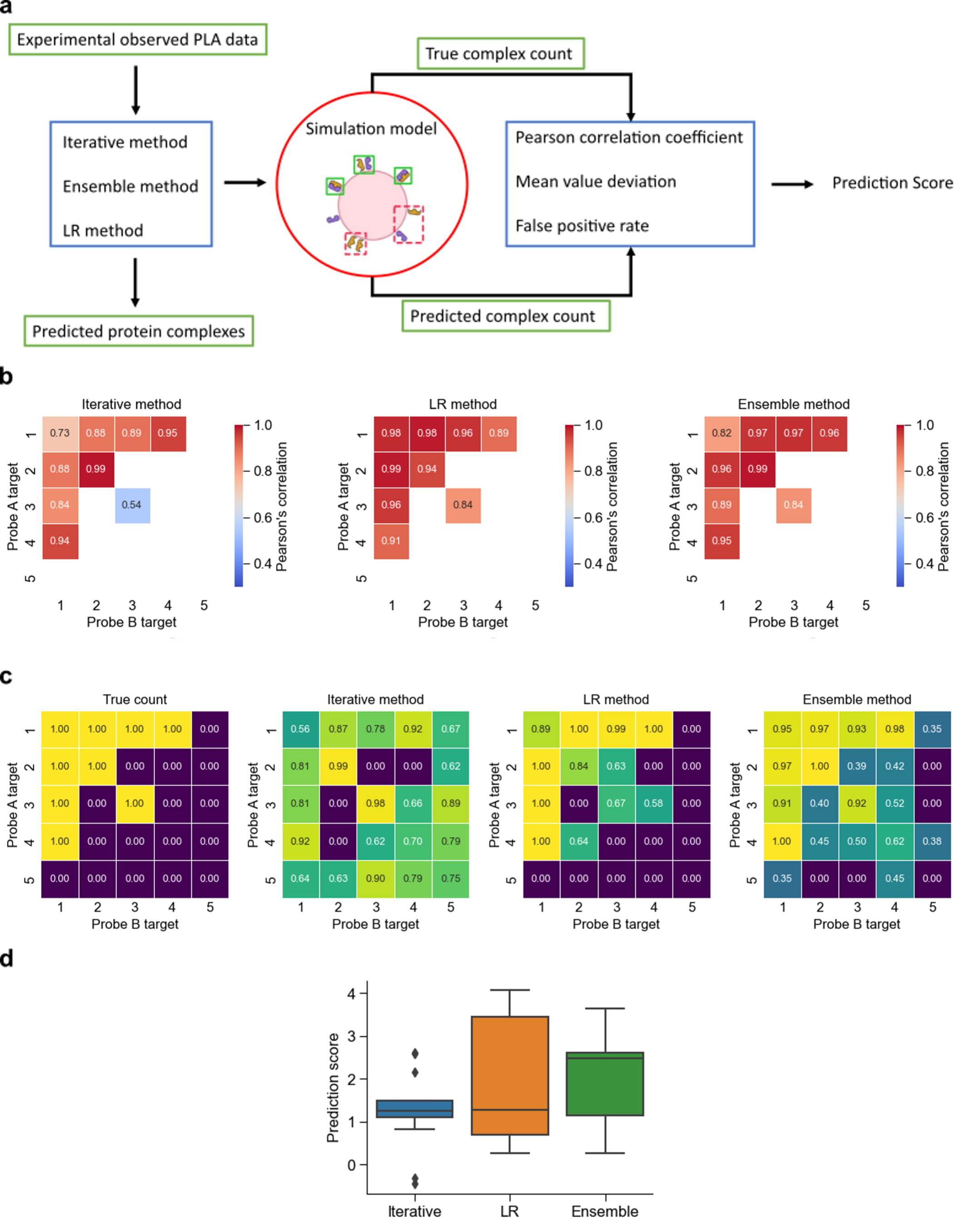

Supplement: S10 Fig — (a) Scheme of using simulation model to evaluate prediction algorithms with a quantitative scoring strategy. (b) The Pearson’s correlation coefficient between true and predicted complex counts for each method. (c) The percent of cells called to have a protein complex for each method, along with the true counts. (d) The median prediction score of the ensemble method is higher than both the LR and iterative when comparing across all scenarios. Ensemble also displays a lower variance than LR. (TIF) [file pcbi.1011915.s010.tif]

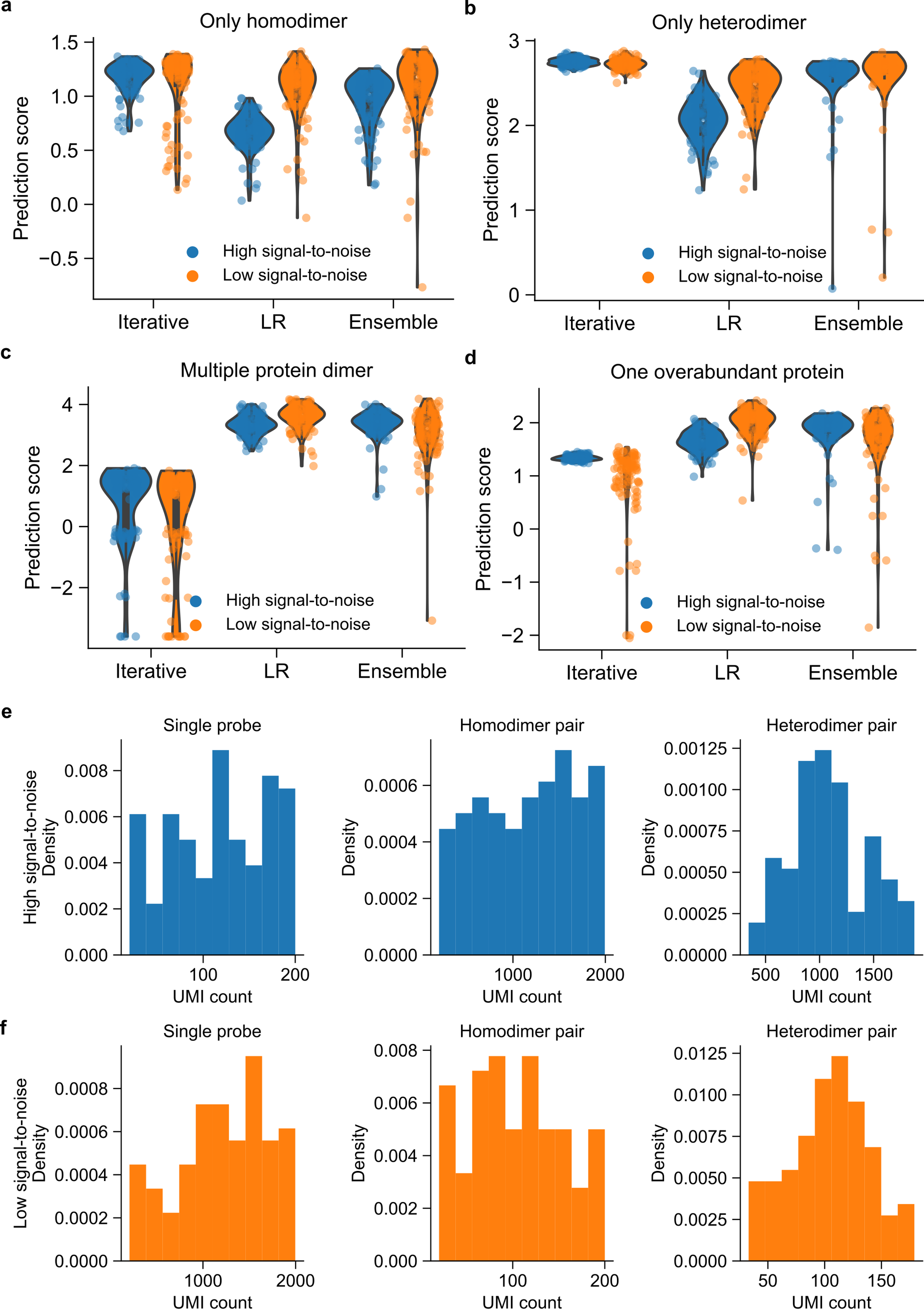

Supplement: S11 Fig — (a-d) Violin plot showing the prediction score of three methods under different simulation scenario with either high signal-to-noise or low signal-to-noise cases. (e) Distribution of input from random generator under high signal-to-noise ratio. Single probe and homodimer pair follow a uniform distribution while heterodimer pair follows a triangular distribution. (f) Distribution of input from random generator under low signal-to-noise ratio. Single probe and homodimer pair follow uniform distribution while heterodimer pair follows triangular distribution. (TIF) [file pcbi.1011915.s011.tif]

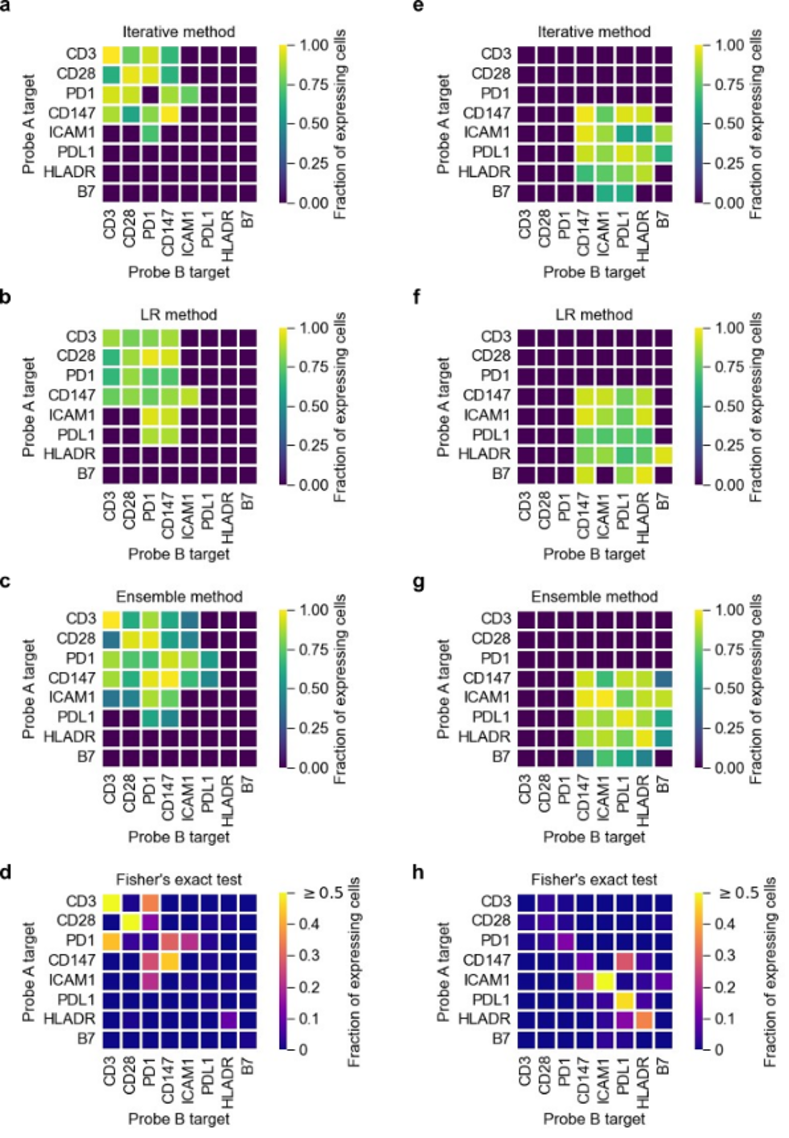

Supplement: S12 Fig — (a-d) Heatmaps showing the fraction of Jurkat cells that express a protein complex, as predicted by (a) the iterative method, (b) the LR method, (c) the Ensemble method, and (d) the Fisher’s exact test. (e-h) Heatmaps showing the fraction of Raji cells that express a protein complex, as predicted by (e) the iterative method, (f) the LR method, (g) the Ensemble method, and (h) the Fisher’s exact test. (TIF) [file pcbi.1011915.s012.tif]

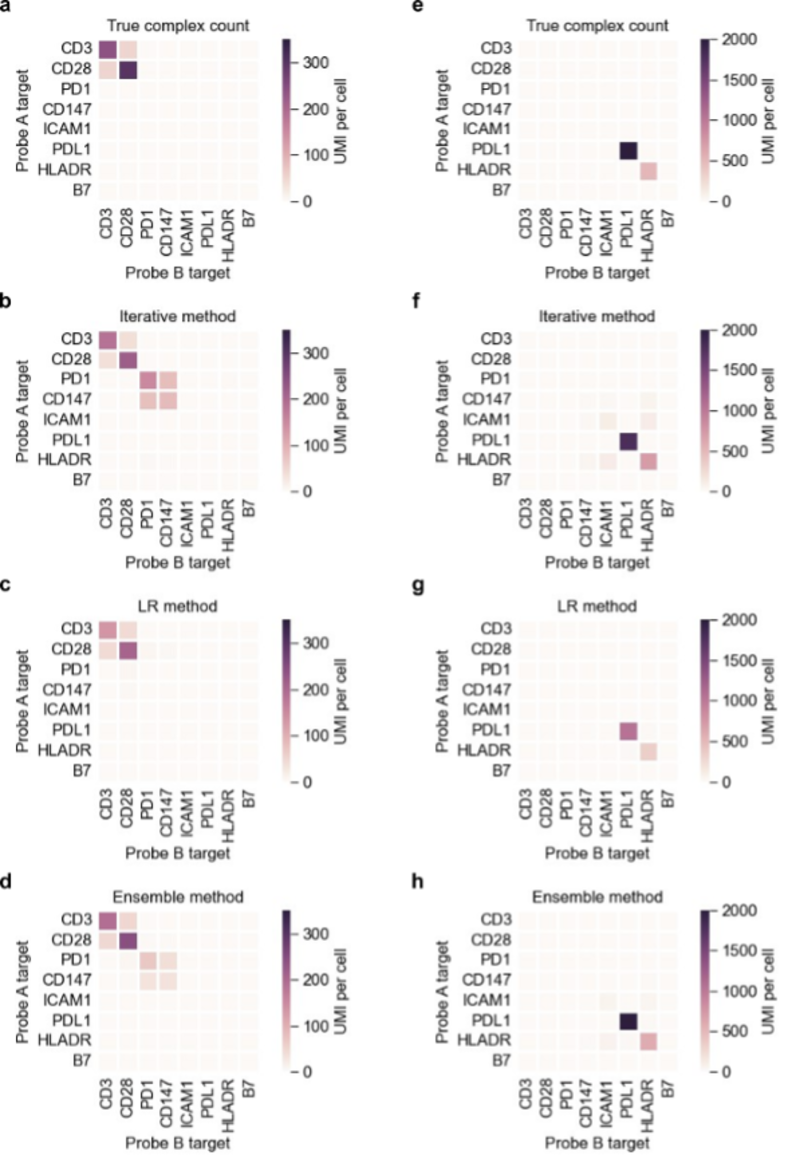

Supplement: S13 Fig — (a-d) Heatmaps of (a) true protein complex counts, and protein complex counts predicted by (b) iterative method, (c) LR method, and (d) Ensemble method in T cell simulation. (e-h) Heatmaps of (e) true protein complex counts, and protein complex counts predicted by (f) iterative method (g) LR method, and (h) the Ensemble method in B cell simulation. Here, the simulation parameters were chosen such that the total protein abundance was similar to experimental data. T cells were simulated to only express the protein complexes CD3:CD3, CD28:CD28, CD3:CD28 and CD28:CD3. B cells were simulated to only express the protein complexes PDL1:PDL1 and HLADR:HLADR. (TIF) [file pcbi.1011915.s013.tif]
