# Supplementary material for: Computational prediction of protein interactions in single cells by proximity sequencing
Source: PLoS Comput Biol. 2024 Mar 14;20(3):e1011915. doi: 10.1371/journal.pcbi.1011915 (PMC10939233; doi:10.1371/journal.pcbi.1011915)
Supplement: S1 Table — (DOCX) [file pcbi.1011915.s014.docx]

| **Parameter** | **Values** | **Note** |
| --- | --- | --- |
| R | 5000 | Cell radius (1 unit = 1 nm) |
| d_ligation_ | 50 | Ligation distance |
| Figures S1, S3, S6 |  |  |
| c_i,j_ | $\left[ \begin{matrix} 100 & 50 & 0 & 0 & 0 \\ 50 & 0 & 0 & 0 & 0 \\ 0 & 0 & 0 & 0 & 0 \\ 0 & 0 & 0 & 0 & 0 \\ 0 & 0 & 0 & 0 & 0 \end{matrix} \right]$ | True protein complex count |
| A_i_ | $\left[ \begin{matrix} 2000 & 1000 & 500 & 200 & 100 \end{matrix} \right]$ | Non-interacting probe A count |
| Bj | $\left[ \begin{matrix} 2000 & 1000 & 500 & 200 & 100 \end{matrix} \right]$ | Non-interacting probe B count |
| Figure 3 |  |  |
| c_i,j_ | $\left[ \begin{matrix} 850 & 750 & 0 \\ 750 & 1400 & 0 \\ 0 & 0 & 0 \end{matrix} \right]$ | True protein complex count |
| A_i_ | $\left[ \begin{matrix} 20 & 15 & 2 \end{matrix} \right]$ | Non-interacting probe A count |
| Bj | $\left[ \begin{matrix} 20 & 15 & 2 \end{matrix} \right]$ | Non-interacting probe B count |
| Figure 4b  c_i,j_  A_i_  Bj | $\left[ \begin{matrix} 3000 & 1500 & 2000 & 1000 & 0 \\ 1500 & 3000 & 0 & 0 & 0 \\ 2000 & 0 & 2000 & 0 & 0 \\ 1000 & 0 & 0 & 0 & 0 \\ 0 & 0 & 0 & 0 & 0 \end{matrix} \right]$  $[\begin{matrix} 30 & 30 & 20 & 10 & 10 \end{matrix}]$  $[\begin{matrix} 30 & 30 & 20 & 10 & 10 \end{matrix}]$ | True protein complex count  Non-interacting probe A count  Non-interacting probe B count |
| Figure 4c  c_i,j_  A_i_  Bj | $\left[ \begin{matrix} 300 & 150 & 200 & 100 & 0 \\ 150 & 300 & 0 & 0 & 0 \\ 200 & 0 & 200 & 0 & 0 \\ 100 & 0 & 0 & 0 & 0 \\ 0 & 0 & 0 & 0 & 0 \end{matrix} \right]$  $[\begin{matrix} 3000 & 1000 & 3000 & 1000 & 1000 \end{matrix}]$    $[\begin{matrix} 3000 & 1000 & 3000 & 1000 & 1000 \end{matrix}]$ | True protein complex count  Non-interacting probe A count  Non-interacting probe B count |
| Figure S2 |  |  |
| c_i,j_ | $\left[ \begin{matrix} 0 & 0 & 0 \\ 0 & 0 & 0 \\ 0 & 0 & 0 \end{matrix} \right]$ | True protein complex count |
| A_i_ | $\left[ \begin{matrix} 2 & 20.1 & 0.6 \end{matrix} \right]$ | Non-interacting probe A count |
| B_j_ | $\left[ \begin{matrix} 5.7 & 41.6 & 0.1 \end{matrix} \right]$ | Non-interacting probe B count |
| Figure S4, S5  c_i,j_  A_i_ & B_j_  c_i,j_  A_i_ & B_j_  c_i,j_  A_i_ & B_j_  c_i,j_  A_i_ & B_j_  P_i_ & P_j_ | Cell type 1  $\left[ \begin{matrix} 2000 & 500 & 0 \\ 500 & 0 & 0 \\ 0 & 0 & 0 \end{matrix} \right]$  $\left[ \begin{matrix} 2500 & 2000 & 4000 \end{matrix} \right]$  Cell type 2  $\left[ \begin{matrix} 0 & 0 & 0 \\ 0 & 0 & 2000 \\ 0 & 2000 & 0 \end{matrix} \right]$  $\left[ \begin{matrix} 5000 & 500 & 2000 \end{matrix} \right]$  Cell type 3  $\left[ \begin{matrix} 3000 & 0 & 1000 \\ 0 & 0 & 0 \\ 1000 & 0 & 0 \end{matrix} \right]$  $\left[ \begin{matrix} 1000 & 2500 & 3000 \end{matrix} \right]$  (Figure S4a)  $\left[ \begin{matrix} 3000 & 0 & 1000 \\ 0 & 0 & 0 \\ 1000 & 0 & 0 \end{matrix} \right]$  $\left[ \begin{matrix} 1000 & 50 & 500 \end{matrix} \right]$  $\left[ \begin{matrix} 0.2 & 0.1 & 0.05 \end{matrix} \right]$ | True protein complex count  Non-interacting probe A/B count  True protein complex count  Non-interacting probe A/B count  True protein complex count  Non-interacting probe A/B count  True protein complex count  Non-interacting probe A/B count  Nonspecific binding probability of each antibody in both panels |
| Figure S7a |  |  |
| c_i,j_ | $\left[ \begin{matrix} 0 & 0 & 0 \\ 0 & 0 & 0 \\ 0 & 0 & 0 \end{matrix} \right]$ | True protein complex count |
| A_i_ | $\left[ \begin{matrix} 1000 & 1000 & 100 \end{matrix} \right]$ | Non-interacting probe A count |
| B_j_ | $\left[ \begin{matrix} 1000 & 1000 & 100 \end{matrix} \right]$ | Non-interacting probe B count |
| Figure S7b |  |  |
| c_i,j_ | $\left[ \begin{matrix} 200 & 0 & 0 \\ 0 & 0 & 0 \\ 0 & 0 & 0 \end{matrix} \right]$ | True protein complex count |
| A_i_ | $\left[ \begin{matrix} 1000 & 1000 & 100 \end{matrix} \right]$ | Non-interacting probe A count |
| B_j_ | $\left[ \begin{matrix} 1000 & 1000 & 100 \end{matrix} \right]$ | Non-interacting probe B count |
| Figure S12 |  |  |
| c_i,j_ | $\left[ \begin{matrix} 240 & 50 & 0 & 0 & 0 & 0 & 0 & 0 \\ 50 & 300 & 0 & 0 & 0 & 0 & 0 & 0 \\ 0 & 0 & 0 & 0 & 0 & 0 & 0 & 0 \\ 0 & 0 & 0 & 0 & 0 & 0 & 0 & 0 \\ 0 & 0 & 0 & 0 & 0 & 0 & 0 & 0 \\ 0 & 0 & 0 & 0 & 0 & 0 & 0 & 0 \\ 0 & 0 & 0 & 0 & 0 & 0 & 0 & 0 \\ 0 & 0 & 0 & 0 & 0 & 0 & 0 & 0 \end{matrix} \right]$ | True protein complex count |
| A_i_ | $\left[ \begin{matrix} 50 & 330 & 3400 & 2400 & 16 & 3 & 100 \end{matrix} 1] \right.$ | Non-interacting probe A count |
| B_j_ | $\left[ \begin{matrix} 50 & 330 & 3400 & 2400 & 16 & 3 & 100 \end{matrix} 1] \right.$ | Non-interacting probe B count |
| Figure S13 |  |  |
| c_i,j_ | $\left[ \begin{matrix} 0 & 0 & 0 & 0 & 0 & 0 & 0 & 0 \\ 0 & 0 & 0 & 0 & 0 & 0 & 0 & 0 \\ 0 & 0 & 0 & 0 & 0 & 0 & 0 & 0 \\ 0 & 0 & 0 & 0 & 0 & 0 & 0 & 0 \\ 0 & 0 & 0 & 0 & 0 & 0 & 0 & 0 \\ 0 & 0 & 0 & 0 & 0 & 3000 & 0 & 0 \\ 0 & 0 & 0 & 0 & 0 & 0 & 500 & 0 \\ 0 & 0 & 0 & 0 & 0 & 0 & 0 & 0 \end{matrix} \right]$ | True protein complex count |
| A_i_ | $\left[ \begin{matrix} 5 & 60 & 30 & 1000 & 2500 & 3 & 5000 & 4 \end{matrix}] \right.$ | Non-interacting probe A count |
| B_j_ | $\left[ \begin{matrix} 5 & 60 & 30 & 1000 & 2500 & 3 & 5000 & 4 \end{matrix}] \right.$ | Non-interacting probe B count |
