# Supplementary material for: Computational prediction of protein interactions in single cells by proximity sequencing
Source: PLoS Comput Biol. 2024 Mar 14;20(3):e1011915. doi: 10.1371/journal.pcbi.1011915 (PMC10939233; doi:10.1371/journal.pcbi.1011915)
Supplement: S2 Table — (DOCX) [file pcbi.1011915.s015.docx]

| Simulation contexts | | Iterative method | LR method | Ensemble method |
| --- | --- | --- | --- | --- |
| Multiple protein  complexes, 5  proteins | High signal, low noise | 2.15 | 0.82 | 2.54 |
|  | High noise, low signal | 1.49 | 3.45 | 3.06 |
| Multiple protein  complexes,  3 proteins | High signal, low noise | 1.28 | 4.07 | 3.57 |
|  | High noise, low signal | 1.13 | 3.55 | 2.51 |
|  | Similar signal & noise | 1.29 | 3.62 | 3.65 |
| Only homodimers,  3 proteins | High signal, low noise | 1.09 | 0.55 | 1.05 |
|  | High noise, low signal | 1.24 | 1.18 | 1.14 |
| Only heterodimers,  3 proteins | High signal, low noise | 2.61 | 0.70 | 2.48 |
|  | High noise, low signal | 2.59 | 2.54 | 2.60 |
| One over-abundant protein forming complexes, 3 proteins | High signal, low noise | 1.23 | 1.27 | 1.76 |
|  | High noise, low signal | 0.83 | 2.08 | 2.08 |
| One over-abundant protein forming only homodimer, 3 proteins | High signal, low noise | -0.32 | 0.27 | 0.27 |
|  | High noise, low signal | -0.45 | 0.45 | 0.41 |

For simulation parameters of different biological scenario tests to generate prediction score table (S2 Table), please refer to <https://github.com/tay-lab/Prox-seq_computation>.
